# Investigating the aspect of asymmetry in brain-first versus body-first Parkinson’s disease

**DOI:** 10.1038/s41531-024-00685-3

**Published:** 2024-03-30

**Authors:** S. S. Lövdal, G. Carli, B. Orso, M. Biehl, D. Arnaldi, P. Mattioli, A. Janzen, E. Sittig, S. Morbelli, J. Booij, W. H. Oertel, K. L. Leenders, S. K. Meles

**Affiliations:** 1 Department of Nuclear Medicine and Molecular Imaging, University Medical Center Groningen, Groningen, Netherlands; 2 Bernoulli Institute for Mathematics, Computer Science and Artificial Intelligence, University of Groningen, Groningen, Netherlands; 3 Department of Neuroscience, Rehabilitation, Ophthalmology, Genetics, Maternal and Child Health (DINOGMI), University of Genoa, Genoa, Italy; 4 SMQB, Institute of Metabolism and Systems Research, College of Medical and Dental Sciences, University of Birmingham, Birmingham, UK; 5 Neurophysiopathology Unit, IRCCS Ospedale Policlinico S. Martino, Genoa, Italy; 6 Department of Neurology, Philipps-University Marburg, Marburg, Germany; 7 Department of Health Sciences, University of Genoa, Genoa, Italy; 8 Nuclear Medicine Unit, IRCCS Ospedale Policlinico S. Martino, Genoa, Italy; 9 Department of Radiology and Nuclear Medicine, Amsterdam UMC, University of Amsterdam, Amsterdam, Netherlands; 10 Department of Neurology, University Medical Center Groningen, Groningen, Netherlands

**Keywords:** Parkinson's disease, Experimental models of disease

## Abstract

Parkinson’s disease (PD) is characterized by a progressive loss of dopaminergic neurons in the substantia nigra. Recent literature has proposed two subgroups of PD. The “body-first subtype” is associated with a prodrome of isolated REM-sleep Behavior Disorder (iRBD) and a relatively symmetric brain degeneration. The “brain-first subtype” is suggested to have a more asymmetric degeneration and a prodromal stage without RBD. This study aims to investigate the proposed difference in symmetry of the degeneration pattern in the presumed body and brain-first PD subtypes. We analyzed ^123^I-FP-CIT (DAT SPECT) and ^18^F-FDG PET brain imaging in three groups of patients (iRBD, *n* = 20, de novo PD with prodromal RBD, *n* = 22, and de novo PD without RBD, *n* = 16) and evaluated dopaminergic and glucose metabolic symmetry. The RBD status of all patients was confirmed with video-polysomnography. The PD groups did not differ from each other with regard to the relative or absolute asymmetry of DAT uptake in the putamen (*p* = 1.0 and *p* = 0.4, respectively). The patient groups also did not differ from each other with regard to the symmetry of expression of the PD-related metabolic pattern (PDRP) in each hemisphere. The PD groups had no difference in symmetry considering mean FDG uptake in left and right regions of interest and generally had the same degree of symmetry as controls, while the iRBD patients had nine regions with abnormal left–right differences (*p* < 0.001). Our findings do not support the asymmetry aspect of the “body-first” versus “brain-first” hypothesis.

## Introduction

Parkinson’s disease (PD) is a neurodegenerative disease characterized by intracellular inclusions of misfolded *α*-synuclein^1^. The clinical diagnosis of PD is based on the cardinal motor symptoms bradykinesia, rigidity, and/or rest tremor^2^, associated with a critical loss of dopaminergic neurons in the substantia nigra. Degeneration of the nigrostriatal dopaminergic system can be confirmed in vivo by brain imaging with ^123^I-N-*ω*-fluoropropyl-2*β*-carbomethoxy-3*β*-{4-iodophenyl}nortropane (^123^I-FP-CIT) single photon emission computed tomography (SPECT) (dopamine transporter (DAT) SPECT) or with ι-3,4-dihydroxy-6-[^18^F]fluorophenylalanine positron emission tomography (^18^F-FDOPA PET). Patients with PD typically have an asymmetric onset of motor features which start in the bodyside contralateral to the predominant dopaminergic deficit. This asymmetry becomes less prominent over the course of the illness^3^. However, approximately 20% of de novo PD patients have a symmetric disease onset^4^.

In PD, brain changes extend beyond the nigrostriatal dopaminergic pathway and consequently involve several neuronal networks, causing various non-motor symptoms^5^. Functional changes in these networks can be examined using ^18^F-Fluorodeoxyglucose PET (^18^F-FDG PET). ^18^F-FDG PET studies have identified a PD-related pattern (PDRP) characterized by relatively increased glucose metabolism in the pallidum, thalamus, pons and cerebellum, and relative hypometabolism of the premotor cortex, supplementary motor area, and parietal association regions^6^. PDRP expression can be quantified and has been observed to increase with disease progression and decrease with symptomatic treatment^7,8^. Only modest correlations exist between PDRP expression and striatal binding ratios measured by ^18^F-FDOPA PET or DAT SPECT imaging, indicating that the PDRP reflects more widespread functional brain changes^9–11^. In contrast to dopaminergic changes, PDRP expression shows no asymmetry, even in PD patients with strictly unilateral symptoms^12^.

Despite clinical asymmetry being well documented, its origin and role in disease pathology are unclear. Some studies have suggested that the asymmetric onset of PD is not random but is directed by brain lateralization (i.e. hand dominance)^13,14^. Explanations have been sought amongst structural and biochemical hemispheric differences that may cause specific unilateral vulnerabilities for PD pathology^15^.

The issue of asymmetry is a focal point in the recently proposed “brain-/body-first” (BBF) hypothesis by Borghammer and colleagues^16^. This hypothesis delineates two primary subtypes of PD. In the body-first subtype, the aggregation of *α*-synuclein is posited to initiate in the gastrointestinal tract, progressing via the vagus nerve to the brainstem, substantia nigra, and eventually the cerebral cortex. This subtype demonstrates an extended prodromal phase due to the involvement of multiple synapses in the gut-to-brain progression. During this phase, REM sleep behavior disorder (RBD) emerges, coinciding with the involvement of the locus coeruleus/subcoeruleus complex in the brainstem. The presence of RBD prior to motor symptoms or dementia onset is considered a reliable indicator of the body-first subtype. Conversely, the brain-first subtype is proposed to originate unilaterally, for instance, in the amygdala^17^. Notably, this progression involves substantial damage to the substantia nigra before observable damage to the autonomic system or the onset of RBD symptoms. RBD may manifest in later stages of brain-first PD, following the spread of the pathology from one hemisphere to the brainstem (top-down propagation). Borghammer and colleagues introduced and expanded upon the BBF hypothesis, proposing the *α*-Synuclein Origin site and Connectome (SOC) hypothesis in 2021^16,17^. An essential premise of this updated BBF hypothesis is that *α*-synuclein propagation is contingent upon the structural brain connectome. Consequently, the SOC hypothesis seeks to predict and explain the (a)symmetrical presentation of clinical symptoms and dopaminergic degeneration in PD. Specifically, in the body-first subtype, the pathological spread is theorized to be symmetrical, facilitated by the overlapping motor innervation of the right and left dorsal vagal nerves within the gastrointestinal tract. This concept draws support from anatomical evidence in animal studies^18^. Conversely, in the brain-first subtype, the pathology primarily spreads to ipsilateral brain structures, guided by intra-hemispheric brain connections, which account for 90*%* of total brain connections. This pattern results in a more asymmetric propagation of the pathology.

Cross-sectional neuropathological data^19,20^, along with multi-modality in-vivo imaging^21^ have provided support for the BBF hypothesis. However, a limited number of studies have tested the validity of its predictions on asymmetry in distinct clinical cohorts of patients presumed to exhibit brain-first or body-first PD. Using ^18^F-FDOPA PET imaging, Knudsen et al. examined individuals with iRBD alongside de novo PD patients, both with and without RBD. They classified the patients as body-first and brain-first subtypes based on the presence or absence of iRBD. Thus, the iRBD group (*n* = 21) was labeled as prodromal body-first PD. Among PD patients, those with RBD at least two years before the onset of motor symptoms (PDRBD+, *n* = 11) were classified as the body-first subtype, while those without RBD (PDRBD-, *n* = 22) were considered the brain-first subtype^22^. The authors found a more symmetric nigrostriatal degeneration in iRBD and PDRBD+ compared to PDRBD-, in line with the BBF hypothesis. In a similar design, Banwinkler et al. (2022)^23^ used a questionnaire-based assessment of iRBD presence and (a)symmetric nigrostriatal dopaminergic degeneration (measured with ^123^I-FP-CIT SPECT) as proxies to distinguish between brain-first and body-first subtypes in a cohort of 255 de novo PD patients. Their investigation focused on comparing hemispheric gray matter loss between these subtypes. Surprisingly, the study did not uncover discernible patterns of brain atrophy between the presumed brain-first and body-first PD subtypes. Additionally, no correlation was observed between the asymmetry in putaminal dopaminergic denervation and the asymmetry in gray matter volume. These results challenge the notion that the spread of pathology throughout the brain aligns with patterns observed in the dopaminergic system, thus questioning the concept of the spread of pathology based on brain connectome principles.

It is tempting to use a degree of asymmetry as a marker to identify PD subtypes in clinical and research settings. However, first, it is crucial to investigate whether asymmetry is indeed associated with the suggested brain/body-first subtypes. In this retrospective study, we compare the degree of asymmetry in putamen DAT binding (^123^I-FP-CIT SPECT), PDRP expression, regional brain glucose metabolism and motor scores between presumed body-first and brain-first PD. Based on the SOC theory, individuals without RBD, indicating the brain-first subtype (PDRBD-), would exhibit greater asymmetry in putamen DAT binding, hemispheric PDRP expression, and regional glucose metabolism (particularly in the amygdala). Conversely, we anticipate that individuals with RBD as a prodromal phase (body-first subtype, PDRBD+), as well as those presenting with iRBD (presumed prodromal phase of body-first PD), would reveal more symmetric findings in these imaging markers.

## Results

### Patients

Table 1 reports the demographic and clinical characteristics of the patient groups. The RBD status of all patients was confirmed with video-polysomnography. The PDRBD+ group was older than PDRBD- (*p* = 0.03) and HC_*IT*_ (*p* = 0.03). The iRBD patients were younger than both PDRBD+ and PDRBD− (*p* ≤ 0.002 for both). There was no difference in disease duration (defined by the duration of motor symptoms) between the two PD groups (*p* = 0.5). The PDRBD+ group had a median duration of RBD symptoms before the onset of motor symptoms of 2.3 years, with a minimum duration of 0.5 years. There was a significantly higher fraction of males in the iRBD group compared to PDRBD− (*p* = 0.009). PDRBD+ had a similar total UPDRS-III score as PDRBD- (*p* = 0.5).

**Table 1:** Demographic and clinical characteristics of the patient groups

	iRBD	PDRBD+	PDRBD−	HC_NL_	HC_IT_^a^
*n*	20	22	16	49	42
Sex, male %	90	73	44	55	64
Age, years	62.7 (5.1)	74.2 (5.3)	69.1 (6.5)	58.5 (11.6)	69.6 (8.5)
Motor symptoms^b^	–	1 [0.7, 2]	1 [1, 2.1]	–	–
Total RBD dur^c^	5.0 [3.0, 6.0]	3.5 [2.3, 6.5]	–	–	–
Prodr. iRBD dur^d^	–	2.3 [1, 5.5]	–	–	–
PDRP *z*-score	1.3 (1.2)	2.4 (1.6)	0.9 (1.4)	0 (1)	0 (1)
H&Y stage, %	0:100	1:27, 2:68, 3:5	1:25, 2:69, 3:6	–	–
UPDRS-III	2.6 (2.0)	21.9 (10.1)	19.6 (8.3)	–	–
MMSE	–	27.8 (2.6)	29.0 (1.8)	–	–
SCOPA-AUT	–	11.4 (5.8)	10.5 (4.5)	–	–
Constipation, %	–	86	54	–	–

The values are reported as mean (STD) or median [IQR].

^a^The iRBD group and HC_NL_ were included in Marburg and Groningen, and the PD groups and HC_IT_ in Genoa.

^b^Time in years since onset of motor symptoms.

^c^Total duration of (i)RBD symptoms prior to inclusion and ^18^F-FDG PET and DAT SPECT scan (years).

^d^Duration of iRBD symptoms prior to onset of motor symptoms (years).

The PDRBD+ group had a higher PDRP *z*-score than PDRBD- (*p* = 0.006) and iRBD (*p* = 0.02). The iRBD group was not significantly different from the PDRBD- group (*p* = 0.4). All three disease groups had a significantly elevated PDRP expression compared to the healthy controls of the same center (*p* ≤ 0.03).

### Putamen DAT binding

**Fig. 1:**
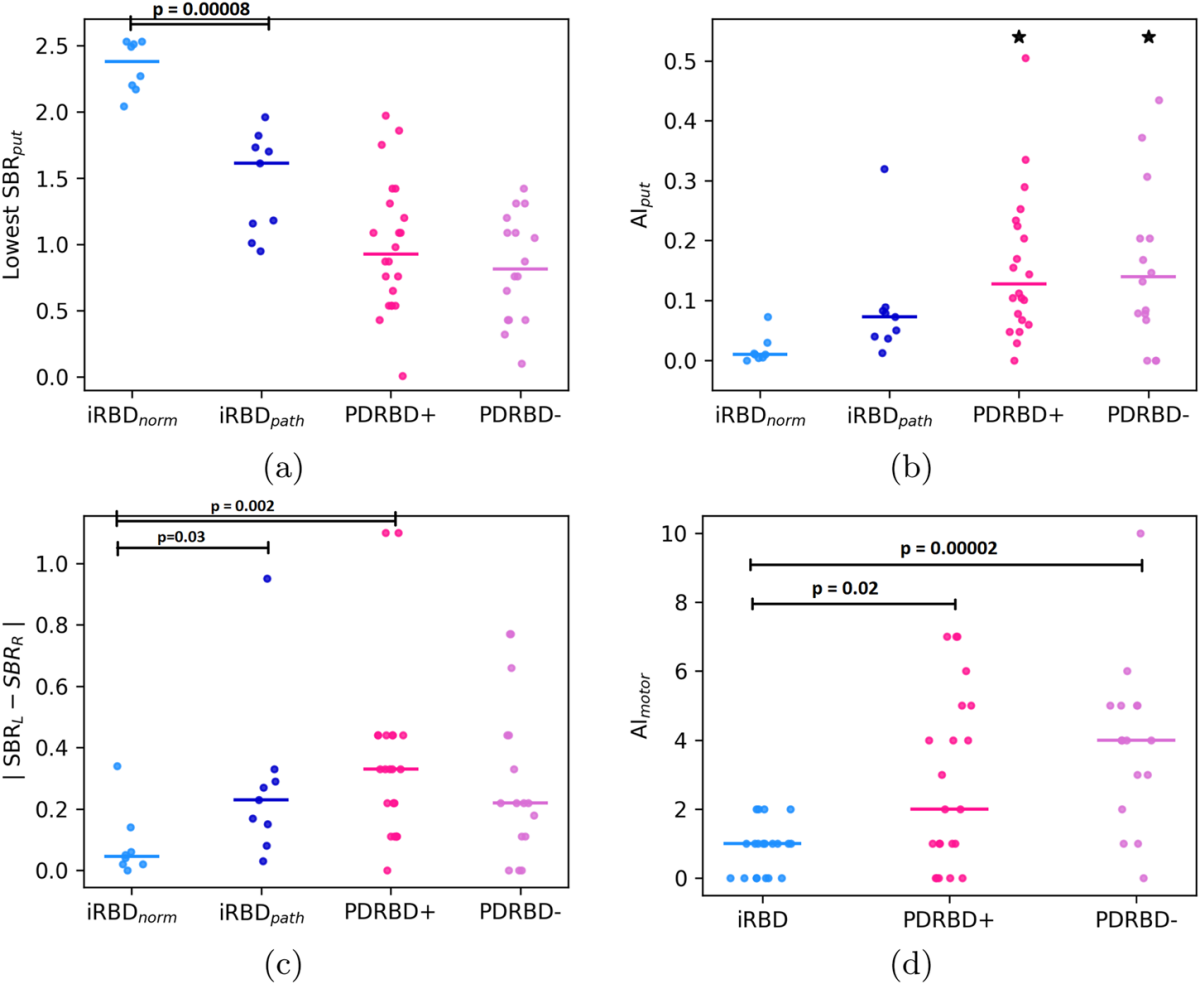
Dopaminergic and motor asymmetry. Lowest putamen SBR (a) and putamen DAT asymmetry index (b) plotted per group, with medians indicated by horizontal bars.One outlier for the putamen in each PD group is indicated by a black star, *AI*_*put*_ = 1.06 for the PDRBD+ and *AI*_*put*_ = 0.69 for the PDRBD− patient. The iRBD patients with an abnormal DAT scan (*n* = 9) have been plotted in dark blue, with the rest of the iRBD patients shown in light blue (*n* = 8). Additionally, **c** shows the absolute difference between left and right putamen SBR, and **d** the difference in left and right UPDRS-III motor scores (here, the total iRBD group, *n* = 20, is shown).

Lowest specific to non-displaceable binding ratio (SBR), putamen (relative) asymmetry index (*AI*_*put*_), and the absolute difference between left and right SBR are shown in Fig. 1. Lowest putamen SBRs were similar in PDRBD+ compared to PDRBD− (*p* = 0.7, median 0.93 vs. 0.82). There was no significant difference in *AI*_*put*_ between PDRBD+ and PDRBD− (*p* = 0.97, median 0.13 vs. 0.14).

17 out of the 20 iRBD patients underwent DAT SPECT imaging, where the group was split based on the DAT scan being evaluated as either normal (iRBD_*norm*_, *n* = 8) or abnormal (iRBD_*path*_, *n* = 9). The median lowest putamen SBR was 2.4 in iRBD_*norm*_ and 1.6 in iRBD_*path*_, with the difference being significant (*p* < 0.0001). We did not directly compare the SBRs between the iRBD and PD groups due to differences in camera and processing pipelines. Furthermore, we did not compare the relative asymmetry index *AI*_*put*_ between groups with significantly (or apparently) differing SBRs. The motivation for this is further treated in the Discussion, see also Supplementary Fig. 3 in the Supplementary Information. Instead, we statistically evaluated the differences in absolute asymmetry.

Considering the absolute difference between the highest and lowest putamen SBR (Figure 1c), the median was 0.33 for PDRBD+, 0.22 for PDRBD-, 0.23 for iRBD_*path*_ and 0.05 for iRBD_*norm*_. There was, again, no significant difference between PDRBD− and PDRBD+ considering this alternative measure for asymmetry (*p* = 0.4). Notably, iRBD_*path*_ was not significantly different from either PDRBD+ (*p* = 0.08) or PDRBD− (*p* = 0.93) with this measure. iRBD_*norm*_ had a significantly lower absolute difference than PDRBD+ (*p* = 0.002) but was not significantly different from PDRBD− (*p* = 0.06) or iRBD_*path*_ after Bonferroni correction (*p* = 0.03).

We used an exploratory cutoff of *AI*_*put*_ = 0.08 to estimate the fraction of patients showing symmetric versus asymmetric degeneration in each group, corresponding to the upper quartile reported in *AI*_*put*_ for HC subjects of the PPMI database in ref. ^22^. Considering this cutoff, all subjects in iRBD_*norm*_ fell below the threshold, while 33% in iRBD_*path*_, 68% in PDRBD+ and 63% in PDRBD− were asymmetric.

We repeated the analysis considering only those PDRBD+ patients with a reported onset of RBD at least two years before the onset of motor symptoms (*n* = 14). This subgroup had a median *AI*_*put*_ of 0.11 and a median lowest putamen SBR of 0.98, none of which were significantly different compared to the PDRBD− group (*p* = 0.9 and 0.8, respectively). There was also no significant correlation between *AI*_*put*_ and duration of RBD symptoms or motor symptoms for any of the PD groups (*p* > 0.2, see Supplementary Fig. 4 of the Supplementary Information).

There was no difference between men and women in *AI*_*put*_, either with the groups considered together or separately (*p* ≥ 0.05). There was a weak correlation between *AI*_*put*_ and age for the PDRBD+ group (*ρ* = 0.51, *p* = 0.02). The absolute difference in left and right putamen SBR was not correlated with age for any group (*p* > 0.1). For PDRBD- and iRBD there was no correlation with age (*p* ≥ 0.3). The absolute difference between left and right putamen SBR did not correlate with the lowest putamen SBR considering the PD patients together (*p* = 0.4), while the relative *AI*_*put*_ correlated with the lowest putamen SBR by *ρ* = −0.63, *p* = 0.00002.

### Hemispheric PDRP expression

**Fig. 2:**
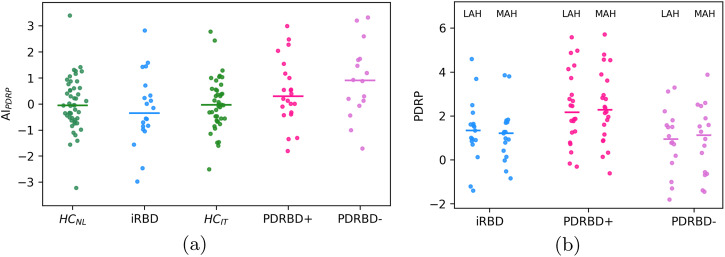
PDRP asymmetry. **a** *z*-scored difference between left and right hemispheric PDRP expression, defined as AI_PDRP_. The median for each group is indicated by a horizontal bar. **b** *z*-scored PDRP expression in the hemisphere with higher and lower putamen DAT binding (least and most affected hemisphere).

There was no significant difference in *AI*_*PDRP*_ between the iRBD, PDRBD+ and PDRBD− groups (*p* ≥ 0.1 for all; see left panel of Fig. 2). Each disease group also did not have a higher value of *AI*_*PDRP*_ than their corresponding HC cohort (*p* > 0.05 for all). Similarly, there were no significant between-group differences in the absolute value of *AI*_*PDRP*_ (*p* ≥ 0.1 for all), emphasizing between-group similarity also in the asymmetric tails of the distributions.

There was no significant difference in PDRP *z*-scores between the most affected hemisphere (MAH, lowest putamen SBR) and the least affected hemisphere (LAH, highest putamen SBR) (*p* > 0.5 for all groups, see Fig. 2, right panel). LAH and MAH PDRP *z*-scores correlated by *ρ* = 0.93, *p* = 1e^−31^.

While most patients had a symmetric PDRP expression in each hemisphere, some could be considered asymmetric: ∣*AI*_*PDPR*_∣ > 2 for 3 iRBD (17%), 4 PDRBD+ (18%), and 3 PDRBD− patients (19%). This corresponded to an absolute difference between LAH and MAH *z*-scored PDRP expression of at least 1.1 for the iRBD patients and 0.8 for the PD patients.

### ^18^F-FDG PET ROI

In PDRBD-, we did not find any regions that systematically deviated from the distribution of *AI*_*ROI*_ in controls. In the amygdala, most subjects had the same degree of symmetry as controls (*p* = 0.3).

We did find significant changes in the degree of (a)symmetry in the PDRBD+ and iRBD groups, in several ROIs, which are indicated in Table 2. In PDRBD+, the amygdala was more asymmetric than in controls (*p* = 0.0004). This could be explained by a higher mean uptake in the right amygdala in PDRBD+ compared to controls (*p* = 0.000002), while the uptake in the left amygdala was similar. PDRBD+ was also more asymmetric than HC in the cerebellum (ROI Cerebellum_10; corresponding to Lobule X of the cerebellar hemisphere, see refs. ^24,25^ for reference). This could mainly be explained by a lower uptake in the right Cerebellum_10 in PDRBD+ compared to HC (*p* = 0.04).

**Table 2:** Summary of ROIs and groups where the asymmetry *z*-score was significantly different from controls

ROI	Group^a^	*AI*_*ROI*_	*p*-value	Type^b^	Primary difference to HC^c^
Amygdala	PDRBD+	−1.10	0.0004	asym	Higher uptake in right
Caudate	iRBD	−1.09	0.00003	asym	Lower uptake in left
Cerebellum_10	PDRBD+	0.87	0.0002	asym	Lower uptake in right
Frontal_Inf_Oper	iRBD	−1.07	0.0003	sym	Lower uptake in left
Frontal_Inf_Tri	iRBD	−0.77	0.0007	sym	Lower uptake in left
Hippocampus	iRBD	−0.76	0.0005	sym	Higher uptake in right
Postcentral	iRBD	−1.07	0.000004	sym	Higher uptake in right
Precentral	iRBD	−1.15	0.00005	sym	Left lower, right higher
Rolandic_Oper	iRBD	−1.40	0.00009	sym	Lower uptake in left
SupraMarginal	iRBD	−0.90	0.00005	sym	Lower uptake in left
Temporal_Mid	iRBD	−1.38	0.00000001	sym	Lower uptake in left

^a^The group is indicated together with its corresponding mean *AI*_*ROI*_, where the mean for controls is always 0 ± 1. The group was either more asymmetric than controls or more symmetric.

^b^Type indicates whether the group was more symmetric or more asymmetric than controls in this region.

^c^Main reasons behind the significant asymmetry. This can be read as “PDRBD+ was more asymmetric than HC in the amygdala, primarily due to having a higher uptake in the right amygdala compared to controls”.

iRBD was significantly different from HC in nine regions. The group was more asymmetric than HC in the caudate nucleus and more symmetric than HC in the inferior frontal gyrus (opercular and triangular part), hippocampus, postcentral gyrus, precentral gyrus, rolandic operculum, supramarginal gyrus and middle temporal gyrus.

No significant differences in *AI*_*ROI*_ were found between PDRBD+ and PDRBD−. However, PDRBD+ was more asymmetric than iRBD in Cerebellum_10, again due to PDRBD+ having a lower uptake on the right side. iRBD was more symmetric than PDRBD+ in the precentral and postcentral gyrus and frontal inferior operculum. iRBD was significantly more symmetric in the supramarginal and middle temporal gyrus compared to both PD groups, respectively (*p* < 0.001). It is important to note that both PDRBD+ and PDRBD− resembled controls regarding symmetry in the above mentioned regions. Thus, the iRBD group showed a deviating distribution, of which the directionality can be found in Table 2.

For the significant ROIs, histograms of *AI*_*ROI*_ as well as z-scored left and right mean uptake values are displayed in Supplementary Figs. 1 and 2 in the Supplementary Information.

Additionally, we considered the overall left vs right uptake difference. For this measure, there was again no difference between PDRBD+ and PDRBD− (*p* = 0.4), while the iRBD group showed significantly lower uptake in the left hemisphere than in the right (*p* = 0.0006 compared to HC). The full results are included in the Supplementary Information (see Supplementary Fig. 8).

### Motor asymmetry

The distributions of motor asymmetry index *AI*_*motor*_*,* as described by the absolute difference between left and right scores of the UPDRS-III scale can be seen in Figure 1d. There was no difference in *AI*_*motor*_ between PDRBD+ and PDRBD− (median 2 vs. 4, *p* = 0.3). Both PD groups were more asymmetric than the iRBD patients (*p* < 0.02 for both).

A summary of the asymmetry indices in the disease groups is shown in Table 3.

**Table 3:** Summary of median asymmetry indices in our patient groups

AI	iRBD^a^	PDRBD+	PDRBD−
*AI*_*put*_	0.01 [0.00, 0.02]/0.07 [0.04, 0.08]	0.13 [0.07, 0.23]	0.14 [0.07, 0.23]
∣SBR_L_−SBR_R_∣	0.05 [0.02, 0.08]/0.23 [0.15, 0.29]	0.33 [0.14, 0.44]	0.22 [0.11, 0.44]
∣*AI*_*PDRP*_∣	0.84 [0.50, 1.47]	0.55 [0.34, 1.50]	1.09[0.41, 1.72]
*AI*_*motor*_	1 [0.0, 1.0]	2 [1.0, 4.8]	4 [2.8, 5]

The values are reported as median [IQR].

^a^For the DAT SPECT-related asymmetry measures, the results are reported separately for the iRBD patients with normal versus abnormal DAT scans.

*AI*_*motor*_ correlated with *AI*_*put*_ (*ρ* = 0.36, *p* = 0.007) considering the full set of patients. *AI*_*put*_ did not correlate with *AI*_*PDRP*_, apart from for the PDRBD− group (*ρ* = 0.56, *p* = 0.02), even though this significance seemed to be driven by a single asymmetric outlier. The *p*-value changed to *p* = 0.26 when removing the outlier. The same *p*-value for PDRBD+ was 0.8. *AI*_*put*_ did not correlate significantly with any *AI*_*ROI*_ after the Bonferroni correction.

## Discussion

Our data does not support the hypothesis that degeneration of the dopaminergic system is more symmetric in body-first PD compared to brain-first PD^17^. We studied 22 de novo PD patients with RBD (PDRBD+; confirmed with video-polysomnography) who reported dream-enactment behavior at least six months before the onset of motor symptoms. We also repeated the analysis for a subset of patients (*n* = 14) who reported RBD symptoms at least 2 years before the onset of motor symptoms. According to the SOC theory, these patients belong to the proposed body-first subtype of PD. Sixteen de novo PD patients without RBD were included to represent the proposed brain-first PD subtype (PDRBD−). Asymmetry indices of striatal DAT binding were not significantly different between the body-first (PDRBD+) and brain-first (PDRBD−) groups. In fact, the majority of PD patients (roughly two-thirds) had asymmetric putamen DAT binding, irrespective of RBD status.

Individuals with iRBD are considered to have a prodromal stage of body-first Lewy body disease. According to the SOC-theory^16,17^, iRBD patients should have symmetric degeneration of the nigrostriatal system. In our study, nine out of 17 iRBD patients had an abnormal DAT scan (their putamen DAT binding ratio was 2 or more standard deviations below age-matched control values). At the group level, these nine iRBD subjects were not different from PDRBD+ or PDRBD− in terms of the absolute difference between left and right putamen SBR. Moreover, three had clearly asymmetric DAT scans (*AI*_*put*_ > 0.08), and one subject in particular showed very asymmetric putamen DAT binding (*AI*_*put*_ = 0.34, which corresponds to the top 20% of asymmetric PD patients in our cohort).

Our findings contradict the results from the study by Knudsen et al.^22^, who found significantly more asymmetry in their PD^−RBD^ (*n* = 22) versus their PD^+RBD^ (*n* = 11) group, using ^18^F-FDOPA PET. The authors also found that DAT SPECT scans in iRBD patients (*n* = 25) were significantly more symmetric compared to de novo PD (*n* = 419, no distinction between subtypes) from the PPMI database. There are a few plausible explanations for the difference in findings between the two studies. First, a possible contributing factor is the difference in sample size. Considering the substantial spread we observed in *AI*_*put*_ for both our PD groups, small sample sizes may not be sufficiently representative of the whole population. Second, Knudsen et al. combined their 11 PD^+RBD^ patients with 12 iRBD patients with a pathological ^18^F-FDOPA PET scan to increase the sample size, forming a combined RBD+ group. This could also have artificially decreased the mean *AI*_*put*_ in their RBD+ group, as illustrated by a decrease in median *AI*_*put*_ compared to PD^+RBD^, and an increase in *p*-value from *p* = 0.001 when comparing RBD+ to PD^−RBD^ to *p* = 0.049 when comparing PD^+RBD^ to PD^−RBD^.

Third, it can be noted that the DAT asymmetry index *AI*_*put*_, being a relative index, features exponential growth with lower SBR (explaining the correlation we observed between *AI*_*put*_ and lowest putamen SBR). This advises caution in its interpretation. Consider two patients with an identical absolute difference in putamen SBR. The patient with overall lower binding ratios will receive a higher *AI*_*put*_ compared to the patient with overall higher binding ratios. We illustrate this in Supplementary Fig. 3 in the Supplementary Information. In the study by Knudsen et al.^22^, mean putamen SBR was significantly lower in the PD^−RBD^ group compared to the PD^+RBD^ group. This could have overestimated *AI*_*put*_ in their PD^−RBD^ group, compared to considering an absolute difference. Therefore, we additionally decided to consider the absolute difference between left and right for putamen SBRs. This measure, as well, showed no between-group differences for our PD groups.

Additionally, differences in clinical characteristics between the two studies should be considered. Our PDRBD+ patients exhibited a shorter total RBD duration, ranging from IQR = [2.3, 6.5] years, compared to Knudsen’s cohort, IQR = [4.8, 16.3] years. A shorter duration of (prodromal) RBD symptoms could imply that some of our PDRBD+ patients might have experienced RBD symptoms and early motor changes more or less simultaneously. This makes the body-first status of our PDRBD+ patients less certain. That being said, we also repeated the analysis for a subset of patients (*n* = 14) who reported RBD symptoms at least 2 years before the onset of motor symptoms, and our results did not change. Additionally, there was no correlation between total RBD duration and *AI*_*put*_ for the PDRBD+ group (see Supplementary Fig. 4). We emphasize that in our PDRBD− group, in which the absence of RBD was confirmed carefully with video-PSG, we also observed cases with symmetric dopaminergic degeneration. We believe that this reinforces our conclusion.

Furthermore, the median duration of motor symptoms was 12 months for both our PDRBD+ and PDRBD− groups, while it was 17 and 21.5 months, respectively, for Knudsen’s^22^. Studies show that dopaminergic asymmetry becomes less prominent as the disease progresses^3,26,27^. Here, Cao et al. found that the degeneration in subregions of the striatum was faster in PD patients with probable RBD, compared to PD without probable RBD, except for in the most affected side of the putamen. This implies that if the putaminergic asymmetry would be similar around the time of onset of motor symptoms, PD patients with RBD possibly progress faster into a relatively more symmetric state. Still, a difference of less than a year in the duration of motor symptoms most likely does not have any major effect on the asymmetry analysis between our study and Knudsen’s^22^.

Finally, the two studies used different imaging modalities. In the present study, DAT SPECT images were analyzed automatically by validated software without anatomical co-registration to individual magnetic resonance imaging (MRI) scans. This is in contrast to Knudsen et al., who used high-quality imaging with ^18^F-FDOPA PET and MRI co-registration^22^. The present study may suffer from lower spatial resolution and signal-to-noise ratio (SPECT vs. PET), and a larger partial volume effect (automatic delineation of volumes of interest). However, it seems unlikely that this will largely influence the determination of the asymmetry index. In fact, many DAT SPECT studies over the last 25 years have been able to capture the asymmetric striatal DAT binding, which is typical for the majority of PD patients^28–32^. To illustrate, we have provided examples of individual DAT SPECT scans from both cameras in Supplementary Figs. 5 and 6 in the Supplementary Information.

For both the cohort in Knudsen et al. and ours, the PDRBD+ group was significantly older than PDRBD−. Striatal DAT binding is known to decrease with age^33^, and the lowest putamen SBR was indeed negatively correlated with age in our cohort. *AI*_*put*_ was, in turn also correlated with age for the PDRBD+ group. However, this leads directly from the dependence of *AI*_*put*_ on the lowest putamen SBR. The absolute difference in left and right putamen SBR was not correlated with age, and we, therefore, assume that the difference in age between our PDRBD+ and PDRBD− groups did not have a major effect on the overall dopaminergic asymmetry analysis.

In addition to very specific changes in the nigrostriatal dopaminergic system, we investigated the degree of asymmetry in a more global measure of brain function: cerebral ^18^F-FDG uptake. The radiotracer ^18^F-FDG is analogous to glucose and provides an index for the first step of the cellular glycolytic pathway. This is often considered a proxy for neuronal and synaptic function, but the cellular source of ^18^F-FDG uptake is complex and reflects the metabolic activity of astrocytes and neurons^34^, and perhaps microglia^35^. In neurodegenerative diseases, the changes in ^18^F-FDG brain uptake occur in a disease-specific pattern, following the brain networks that are affected. Such patterns can be identified with spatial covariance analysis, e.g. the PDRP^36^.

Contrary to the local, asymmetric dopamine depletion in the striatum, the PDRP represents broader functional changes in the underlying pathophysiological pathways of PD. We calculated PDRP scores in each hemisphere in our iRBD, PDRBD+, and PDRBD− groups. Because brain-first PD is suggested to have a more asymmetric intra-cerebral pathology propagation in early disease stages, we expected the metabolic consequence of those changes (PDRP expression) to be asymmetric as well. This would be in contrast to body-first PD subjects, which would have a more symmetric magnitude of PDRP expression in both hemispheres. This was not the case. There was no significant difference between presumed brain- and body-first PD subjects in terms of asymmetry in PDRP expression. At the group level, we also did not find any significant differences between the most- and least affected hemisphere (stratified by the lowest- and highest putamen SBR, respectively). These results are in line with the study by Tang et al.^12^, who described symmetric PDRP expression in PD patients in both early (H&Y stage 1) and more advanced (H&Y 2–3) stages. In line with Huang et al.^37^, PDRP expression in our study was also symmetric in the iRBD group. It must be noted, however, that we found a large degree of spread in the data, both in controls and patients. Although most patients had a similar degree of asymmetry as controls, there were subjects in each patient group that had a significantly higher PDRP expression in one hemisphere.

Because the PDRP is a sum score of all voxels, small but relevant asymmetries may have been obscured. We, therefore, also investigated which regions deviated from the natural asymmetry that was present in controls. Contrary to our hypothesis, we did not find any deviating regions in the PDRBD- (brain first) group. We did find regional deviations in the iRBD group and, to a lesser extent, in the PDRBD+ group, but the meaning of these findings is difficult to interpret. The regions that were found (Table 2) are all components of the PDRP network. How and why natural (a)symmetry is lost in these regions is unclear.

Our study can be considered in relationship to recently published results by Zhou et al.^38^. They combined clinical and imaging data and identified two distinct trajectories in PD using a data-driven approach. The obtained trajectories closely resemble the “brain-first" and “body-first" subtypes, showing specific early and advanced manifestations for each, whereas the subtype resembling “body-first" featured RBD as an early symptom. Considering the modest accuracy of the RBDQ-HK questionnaire used, it would be interesting to see a similar (future) study using PSG-based RBD markers. The study found no significant differences in the asymmetry of free water in the basal forebrain, amygdala, entorhinal cortex, and hippocampus or in the integrity of the substantia nigra and locus coeruleus (using neuromelanin-sensitive MRI measures). These results support that the BBF theory effectively explains various clinical features and disease progression stages in PD. However, the enigma of asymmetry in brain pathology remains unresolved, warranting further investigation.

This study has limitations. Our data is cross-sectional and only describes a snapshot of the disease evolution. Longitudinal data is needed to verify whether the asymmetry concept of the SOC theory may hold at a specific disease stage. It is theoretically possible that PDRBD− patients would have been more asymmetric than PDRBD+ cases had both groups been scanned at an earlier disease stage. Longitudinal data of ^18^F-FDG PET and DAT SPECT scans will become available in an ongoing longitudinal study of RBD patients^39^. However, the prodromal stage of PDRBD− is ill-defined, and it would be challenging to identify such subjects in the general population. This study also shows that studying asymmetry in neuroimaging data is challenging. In the previous paragraph, we have indicated that relative asymmetry indices are nonlinear and should probably not be compared between groups with differing degrees of degeneration. Furthermore, there is also a natural asymmetry in ^18^F-FDG uptake in the majority of brain regions in healthy controls. With regard to our healthy control group, we recognize the absence of biomarkers such as amyloid quantification, capable of stratifying healthy controls based on the risk of developing a neurodegenerative disease. Future studies should consider incorporating such measures to exclude controls that might be in a prodromal phase of neurodegenerative disease, thereby mitigating potential bias in the analyses. This is particularly important when biomarkers of nonspecific neurodegeneration, such as ^18^F-FDG, are involved.

Furthermore, we did not have other imaging markers available to further verify the brain or body-first status of our PD subjects due to the retrospective nature of this study^21^. An important example is ^123^I-metaiodobenzylguanidine (MIBG) scintigraphy, which assesses the sympathetic nervous system. Reduced MIBG uptake in the heart confirms the involvement of the autonomic nervous system (i.e. ’the body’). We assessed autonomic symptoms using the SCOPA-AUT questionnaire and found no significant differences between the two groups, consistent with previous studies^21^. This reinforces the importance of biologically quantifying the peripheral nature of the degeneration (e.g. MIBG scintigraphy).

Finally, we consider that iRBD subjects have prodromal PD or DLB. However, a minority of iRBD patients (<5%) develop multiple systems atrophy (MSA)^40^. Theoretically, our iRBD cohort could include MSA cases. Here we presented the baseline data of our iRBD cohort as a reference^36^. To date, none of our patients have developed MSA, but longer-term follow-up would be needed to substantiate that all our iRBD patients indeed had prodromal Lewy-body disease. Given the small percentage of MSA-converters, we do not believe that the inclusion of MSA cases would significantly alter the results.

It should be noted that iRBD patients develop PD or DLB in approximately equal numbers^40^. In turn, most DLB patients suffer from RBD, and they typically have abnormal MIBG scans and more severe autonomic dysregulation. Therefore, Borghammer et al. posit that DLB and PD are fundamentally the same diseases and that DLB patients fall in the body-first PD category^16^, and further suggest a role for Alzheimer’s co-pathology to understand different subtypes within the brain-first/body-first framework^20^. Two studies have shown that dopaminergic degeneration is indeed more symmetric in DLB compared to PD^41,42^, but these subjects did not confirm body/brain-first status with PSG or other markers. Whether this is indeed related to the routes of propagation of pathology, as suggested by the SOC-theory, remains to be investigated. Results of a study by Arnaldi et al. call this into question^43^. They indicate that patients with iRBD and asymmetric DAT binding in the caudate nucleus had a higher risk of developing DLB, whereas those with more symmetric caudate nucleus DAT binding were at a higher risk of developing PD.

It remains unclear what aspects of asymmetry are important, how they relate to PD pathology, and how we can meaningfully interpret numerical indices of asymmetry. This study found no relationship between *AI*_*put*_ and age, sex, duration of RBD or motor symptoms, or absolute difference between left and right and lowest putamen SBR. It must also be noted that presynaptic dopaminergic imaging and cerebral glucose metabolism are not direct markers of the underlying pathology (i.e. *α*-synuclein aggregation) but reflect the functional brain changes in response to pathology.

In summary, our results show that there is no significant difference in asymmetry between presumed brain-first and body-first PD in terms of presynaptic dopaminergic imaging and cerebral glucose metabolism. Dopaminergic changes are asymmetric in most patients (roughly two-thirds) but symmetric in some. PDRP expression is bilateral in most iRBD, PDRBD+ and PDRBD− patients, but some cases are asymmetric. The SOC hypothesis is compelling and may provide a framework for the pathophysiology of PD. It should be emphasized that our data do not falsify this model but call into question the assumption that body-first PD is more symmetric than brain-first PD. The most important implication of this study is that the degree of (a)symmetry on presynaptic dopaminergic imaging alone should not be used as a proxy to classify patients as brain- or body-first PD in future studies.

## Methods

### Participants

In this retrospective study, we gathered and analyzed clinical and imaging data previously obtained for PD and iRBD patients. These data were collected within the framework of clinical practice (PD) and other specific studies (iRBD)^44,45^.

#### iRBD patients

Twenty patients with iRBD (confirmed with video-polysomnography^46^) were included in the study (age 62.7 ± 5.1 years, 2 females, 18 males) at the University Medical Centre Groningen (UMCG) (*n* = 3) and the Philipps-Universität Marburg, Germany (*n* = 17). They all underwent ^18^F-FDG PET brain imaging at the UMCG around the time of iRBD diagnosis. In the 17 German patients, ^123^I-FP-CIT SPECT was also performed. All patients were evaluated with motor, cognitive, and olfactory testing (Sniffin Sticks: identification subscore). The details of this cohort are provided elsewhere^36,44^.

#### PD patients

We included 38 de novo PD patients from the IRCCS Ospedale Policlinico San Martino in Genoa ^45^. The diagnosis was made following current clinical criteria^2^ and confirmed by a presynaptic dopaminergic deficit on ^123^I-FP-CIT SPECT at baseline and at least two years of clinical follow-up by a movement disorders specialist. All patients underwent video-polysomnography to determine the presence of RBD^47^. The patients underwent a clinical interview at baseline and were evaluated by a sleep neurologist through a semi-structured interview and the assessment of REM Sleep Behavior Disorder Single-Question Screen^48^ to infer the duration of RBD symptoms prior to the onset of motor symptoms. To account for the potential scenario where brain-first PD patients might later develop RBD during the disease progression, we exclusively included PDRBD+ patients who had exhibited RBD symptoms for a longer duration than their motor symptoms. Out of 38 PD patients, 22 had RBD (PDRBD+, age 74.2 ± 5.3, 6 females, 16 males) at least six months before the onset of motor symptoms, and 16 did not have RBD at all (PDRBD−, age 69.1 ± 6.5, 9 females, 7 males). All patients also underwent ^18^F-FDG PET scanning at baseline.

#### HC

For reference, we included ^18^F-FDG PET scans of *n* = 49 healthy controls acquired at the UMCG (HC_NL_, age 58.5 ± 11.6 years, 27 males, 22 females) and *n* = 42 healthy controls at IRCCS Ospedale Policlinico San Martino (HC_IT_, age 69.6 ± 8.5 years, 27 males, 15 females). These subjects did not have a history of neurological or psychiatric disease or other chronic illnesses and were not taking psychoactive medication.

All participants gave their written consent to the study. The study protocol met the approval of the local Ethics Committee, and all participants signed an informed consent form in compliance with the Helsinki Declaration of 1975.

### ^123^I-FP-CIT SPECT acquisition, pre-processing, and analyses

^123^I-FP-CIT SPECT (DAT SPECT) data of iRBD patients were acquired on a dual-head gamma camera with a low-energy high-resolution collimator (Siemens, Symbia). ^123^I-FP-CIT binding in striatal regions was quantified with The Brain Registration & Analysis Software Suite (BRASS™, HERMES Medical, Sweden). Specific to non-specific binding ratios were calculated in the putamen bilaterally, using the occipital cortex as a reference. Binding ratios that were two or more standard deviations lower than age-matched expected control values were considered abnormal (see ref. ^36^ for further details). We divided the iRBD group into those with abnormal DAT binding (most affected putamen > 2 SD below normal) and those with a normal DAT scan.

^123^I-FP-CIT SPECT data of PD patients were acquired using a 2-headed Millennium VG camera (G.E. Healthcare). The reconstructed ^123^I-FP-CIT SPECT images were processed using the BasGan software version 2 based on a high-definition, 3D striatal template derived from Talairach’s atlas. Partial volume effect (PVE) correction is included in the uptake computation of caudate, putamen, and the occipital region background.

For both iRBD and PD patients, SBR) values were calculated according to (putamen–occipital)/occipital.

Similarly to previous studies^4,22^, we obtain an AI according to the relative difference in SBR

1 $${ {A{I}}}_{ {{put}}}=| ({ {SB{R}}}_{ {{R}}}-{ {SB{R}}}_{ {{L}}})/({ {SB{R}}}_{ {{R}}}+{ {SB{R}}}_{ {{L}}})|$$

Additionally, we consider the absolute difference between left and right putamen SBRs: ∣SBR_R_−SBR_L_∣. For our exploratory analysis of the fraction of symmetric versus asymmetric patients, we have used the cutoff value *AI*_*put*_ = 0.08. This corresponds to the upper quartile of the *AI*_*put*_ in DAT scans of healthy controls (*n* = 193, age 60.2 ± 11.3 years) from the PPMI database, as reported by Knudsen et al.^22^. All DAT SPECT data (SBRs and asymmetry indices) included in this paper are based on the directly measured SBRs, and are thus not *z*-scored quantities. The reported DAT SPECT-based measures for asymmetry are absolute values.

Due to the DAT SPECT scans of the iRBD and PD groups being acquired using different cameras and processing pipelines, we refrain from formally comparing the SBRs obtained at different medical centers. We assume that relative and absolute measures of asymmetry are not significantly affected by the above-mentioned differences.

### ^18^F-FDG PET acquisition, pre-processing and analyses

^18^F-FDG PET scans of 20 iRBD patients and 49 HC were performed on a Siemens Biograph mCT64 or mCT40 PET/CT camera (Siemens, Munich, Germany) at the UMCG. Images were reconstructed with time-of-flight, point-spread function (3 iterations, 21 subsets) and smoothed with a Gaussian 8-mm full-width-at-half-maximum spatial filter. The matrix size was 256 (corresponding to a voxel size of 2.00 × 3.18 × 3.18 mm^3^). Central nervous system depressants and any RBD-related medications (i.e., melatonin or clonazepam) were discontinued in all subjects for at least 24 h before scanning.

^18^F-FDG PET scans of 38 PD patients and 42 HC were performed on an SIEMENS Biograph 16 PET/CT hybrid system with a total axial field of view of 15 cm and no interplane gap space. Data were reconstructed using the OSEM algorithm (16 subsets and 6 iterations) with a reconstructed voxel size of 1.33 × 1.33 × 2.00 mm^3^.

All scans were spatially normalized to an ^18^F-FDG PET template in the Montreal Neurological Institute (MNI) brain space^49^ using the SPM12 software (Wellcome Centre for Human Neuroimaging, London, UK) implemented in MATLAB (version R2019a; MathWorks, Natick, MA, USA). The iRBD and HC_NL_ acquired at the UMCG were already intrinsically smoothed. We applied the same amount of smoothing to ^18^F-FDG PET scans of PD and HC acquired in Genoa. For the PDRP-related analysis, the smoothed PET images in MNI space underwent intensity normalization using the Scaled Subprofile Model (SSM)^8,50^: masking, log-transformation, subject-demeaning, and grand mean profile-demeaning, based on the space defining reference group used in^51^. To match the symmetric PDRP, the mask we applied consisted of all nonzero voxels in the symmetric PDPR. For the ROI analysis we preprocessed the smoothed images by global mean normalization, using the regions included in the AAL atlas as a mask^24^.

#### PDRP expression

The PDRP is a topographical map reflecting the metabolic pattern in PD. A PDRP score is calculated by computing the dot product between each corresponding voxel in the disease pattern and an ^18^F-FDG PET patient scan, essentially reflecting the degree of similarity between the patient image and the PDRP. In this work, we have used the PDRP as defined in^51^ or equivalent to the Dutch PDRP in^52^. The PDRP was used (without any adjustments) to compute the PDRP scores reported in Table 1, according to

$${ {PDRP}}={ {DP}}\cdot X$$

corresponding to the dot product between each voxel in the disease pattern (*DP*) and the SSM-preprocessed patient scan (*X*). PDRP *z*-scores were then obtained by *z*-scoring each raw value to the mean and standard deviation of the HCs from the same medical center.

#### Hemispheric PDRP expression

The PDRP itself is not completely symmetric by a mathematical definition. To avoid having to correct for asymmetries in the PDRP itself when considering left and right hemispheric PDRP scores, we created a symmetric version of the PDRP by averaging corresponding voxels in the left and right hemispheres. The original and symmetric version can be seen in the Supplementary Information, Supplementary Fig. 7, where the analytic motivation for creating a symmetric disease pattern is also included (see Supplementary notes).

The PDRP score for the left hemisphere was computed according to

$${ {PDR{P}}}_{ {{L}}}={ {D{P}}}_{ {{L}}}\cdot {X}_{ {{L}}}$$

corresponding to the dot product between the voxels in the left hemisphere of the symmetric disease pattern DP_L_, and the voxels in the left hemisphere of a preprocessed patient scan (*X*_L_). The PDRP score for the right hemisphere (PDRP_R_) was computed similarly. We consider the raw difference in PDRP as Δ_PDRP_ = PDRP_L_−PDRP_R_.

We define an asymmetry index (AI) for the PDRP according to Eq (2). Here we consider the difference between the left and the right PDRP score (Δ_PDRP_) and *z*-score it to the difference we observe in HC of the same center:

2 $${ {A{I}}}_{ {{PDRP}}}=\frac{{\Delta }_{{ {PDRP}}}-{\mu }_{{\Delta }_{{ {PDRP,HC}}}}}{{\sigma }_{{\Delta }_{{ {PDRP,HC}}}}}$$

One could consider either the absolute value of *AI*_*PDRP*_ (discarding laterality, only considering whether one side is more affected than the other) or *AI*_*PDRP*_ as we define it above (also taking into account whether a specific hemisphere may be more affected). We statistically compare the between-group differences in both *AI*_*PDRP*_ and ∣*AI*_*PDRP*_∣.

We also analyzed the hemispheric PDRP expression by grouping them according to the hemisphere with the highest (least affected hemisphere, LAH) and lowest putamen DAT binding (most affected hemisphere, MAH). We *z*-scored each PDRP_L_ and PDRP_R_ according to the average hemispheric PDRP expression in HC of the same center.

Original PDRP scores and scores obtained by the symmetric PDRP correlated by *ρ* = 0.997, *p* = e−113 in the UMCG cohort, indicating that the symmetric PDRP was still highly representative of the original pattern. The original and symmetric version of the PDRP can be seen in the Supplementary Information in Supplementary Fig. 7. Additionally, we provide a formal mathematical motivation for using a symmetric version of the PDRP.

#### Regional analysis

We extracted mean uptake values from the pre-processed scans using the 116 regions of the automated anatomical labeling (AAL) atlas^24^, of which 108 regions have a contralateral counterpart. Again, we compute the difference in mean uptake between left and right (Δ_ROI_ = *μ*_L_−*μ*_R_) and *z*-score the difference to the difference in controls according to Eq. (3) for each ROI.

3 $${ {A{I}}}_{ {{ROI}}}=\frac{{\Delta }_{ {{ROI}}}-{\mu }_{{\Delta }_{{ {ROI,HC}}}}}{{\sigma }_{{\Delta }_{{ {ROI,HC}}}}}$$

As we noted that there was often a naturally occurring asymmetry in controls, with one region having consistently higher uptake than the other, it became important to preserve information regarding laterality when considering the ROI asymmetry index. In other words, the raw left and right differences were rarely centered around zero for HC. Therefore it was essential to not consider the absolute value of *AI*_*ROI*_ but to statistically compare the distributions with the sign preserved.

The physiological asymmetry of the human brain makes the interpretation of the ROI asymmetry index more complex, as deviations from the norm can lead to both more asymmetric and more symmetric patterns. Some subjects may exhibit greater asymmetry than the control group. This could be due to either the downregulation of an ROI with a naturally lower uptake or the upregulation of an ROI with a naturally higher uptake. Other subjects may display increased symmetry compared to the control group. This can happen when the naturally higher ROI experiences downregulation or when the naturally lower ROI undergoes upregulation, resulting in an abnormally symmetric pattern. The interpretation of the significant ROIs in Table 2 have been obtained by a visual inspection of the two-dimensional distributions of left and right mean uptake values for each ROI: first, inspecting which side has higher or lower uptake in controls, and then evaluating whether the patient group falls closer to or further away from the mathematically symmetric case (*μ*_L_ = *μ*_R_) compared to controls. The primary reason for the deviating *AI*_*ROI*_ was interpreted in a similar manner.

In simple terms, both AI_PDRP_ and *AI*_*ROI*_ are the *z*-scored difference between the left and right hemispheres, where we preserve the sign of the *z*-score. It is well known that ^18^F-FDG PET scans may suffer from center-specific effects^53^. We therefore *z*-score each Δ_PDRP_ using the distribution of Δ_PDRP_ in HCs from the same center (and similarly for Δ_ROI_). Hereby, the ^18^F-FDG-PET-based quantities are harmonized between the two centers, and the resulting *z*-scores can be statistically compared.

#### Overall hemispheric uptake

We computed the difference in overall left and right activity by considering all left regions of the AAL atlas together, as well as all right regions together. We then measured the difference in global activity per hemisphere by *z*-scoring the L−R uptake difference to HCs from the same medical center. These results can be viewed in Supplementary Fig. 8 in the Supplementary Information. The same preprocessing as for the regional analysis was used.

In addition, the degree of motor symptom asymmetry (*AI*_*motor*_ was calculated similarly to^22^ using the absolute difference between left and right lateralized items of the Movement Disorder Society-Unified Parkinson’s Disease Rating Scale (MDS-UPDRS) Part III:

4 $${ {A{I}}}_{ {{motor}}}=| ({{{\mbox{UPDRS-III}}}}_{ {{R}}}-{{{\mbox{UPDRS-III}}}}_{{ {L}}})|$$

### Statistical analysis

Statistical analysis was performed using Python’s scipy package (version 1.9.3, https://scipy.org/).

For normally distributed continuous variables, we use the ANOVA *F*-test to compare multiple groups with post-hoc pairwise Welch’s *t*-tests. This was appropriate e.g. to compare demographic and clinical properties of subject groups and mean uptake in ROIs. A Kruskal–Wallis *H*-test was applied for non-normal distributions, as evaluated by Shapiro–Wilk tests. We applied the chi-square test of independence for categorical variables as appropriate. We did not compare the SBR of the iRBD and PD patients directly because of the differences in SPECT camera and processing protocols used for the two cohorts and the lack of HC for each centre.

We used a two-sample Kolmogorov–Smirnov (K–S) test to evaluate the differences between groups (iRBD, PDRBD+, and PDRBD−) for the *AI*_*PDRP*_, *AI*_*ROI*_, *AI*_*SBR*_, and *AI*_*motor*_*.*

We used a paired samples *t*-test to evaluate the difference between MAH and LAH PDRP scores and Pearson correlation to assess the correlation between variables.

We required a Bonferroni corrected significance of *p* < 0.05. We note that this requirement results in a rather strict correction for the ROI analysis since we included 54 left–right region pairs from the AAL atlas, requiring *p* = 0.05/54 = 0.00093 for the significance of the ROI asymmetry distributions.

### Supplementary information

Supplementary Information

## Data Availability

Our data is available upon request. Researchers can contact Sanne Meles (s.k.meles@umcg.nl) concerning the data collected at the University Medical Center Groningen and Dario Arnaldi (dario.arnaldi@gmail.com) for the data collected at the IRCCS Ospedale Policlinico S. Martino.
