# Supplementary material for: Investigating the aspect of asymmetry in brain-first versus body-first Parkinson’s disease
Source: NPJ Parkinsons Dis. 2024 Mar 30;10:74. doi: 10.1038/s41531-024-00685-3 (PMC10981719; doi:10.1038/s41531-024-00685-3)
Supplement: Supplementary file 1 — Supplementary Information [file 41531_2024_685_MOESM1_ESM.pdf]

Supplementary Information

Supplementary notes

The mathematical motivation for using a mathematically symmetric PDRP is covered below.

Consider the potential scenario where the PDRP is asymmetric with the PD-related metabolic expression being twice as high in the left hemisphere as in the right. Mathematically speaking, $p_l = 2p_r$, or each pixel value in the left hemisphere p_l being equivalent to twice the magnitude of the corresponding right pixel p_r (twice the hypermetabolic expression, or twice the hypometabolic).

Then, the raw PDRP score of a PD patient having the exact same metabolic expression as the PDPR, would be, with i iterating over each pixel

$$PDRP_L = \sum_{i=0}^{N}(p_l^i)^2 = \sum_{i=0}^{N}(2p_r^i)^2 = \sum_{i=0}^{N} 4(p_r^i)^2 \tag{1}$$

$$PDRP_R = \sum_{i=0}^{N}(p_r^i)^2 \tag{2}$$

Then, consider a PD patient with the same metabolic expression as the first patient, but flipped, with the right side being more affected instead. For this patient,

$$PDRP_L = \sum_{i=0}^{N} p_l^i p_r^i = \sum_{i=0}^{N}(2p_r^i)p_r^i = \sum_{i=0}^{N} 2(p_r^i)^2 \tag{3}$$

$$PDRP_R = \sum_{i=0}^{N} p_r^i p_l^i = \sum_{i=0}^{N}(2p_r^i)p_r^i = \sum_{i=0}^{N} 2(p_r^i)^2 \tag{4}$$

For the first patient, the left-right difference is $3\sum_{i=0}^{N} p_r^2$, and for the second patient, the left-right difference is 0. Clearly an issue becomes apparent here, as we know that the patients are equally asymmetric, only with opposite hemispheres being dominant. However, only one of the patients obtains a nonzero difference between the left and right PDRP scores.

Additionally, since a HC theoretically would have a similar (low) score in each hemisphere, z-scoring the hemispheric PDRP values (to HCs) would not resolve the issue.

This is the main reason that we defined a mathematically symmetric PDRP: To not have to rely on possibily erroneous assumptions, and to be able to formally evaluate asymmetry.

Supplementary Figures

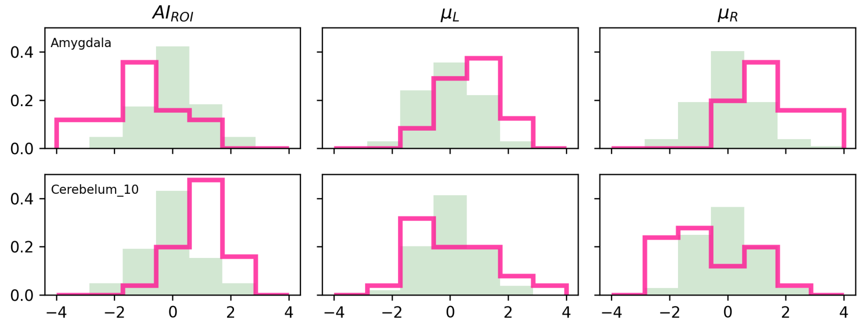

Supplementary Figure 1 ROI asymmetry index (left column, equivalent to the z-scored difference between left and right mean uptake), mean uptake in left (middle column), and mean uptake in the right amygdala and cerebelum_10, respectively. These regions were significantly more asymmetric in PDRBD+, plotted as the pink unfilled histogram, versus controls (solid green histogram).

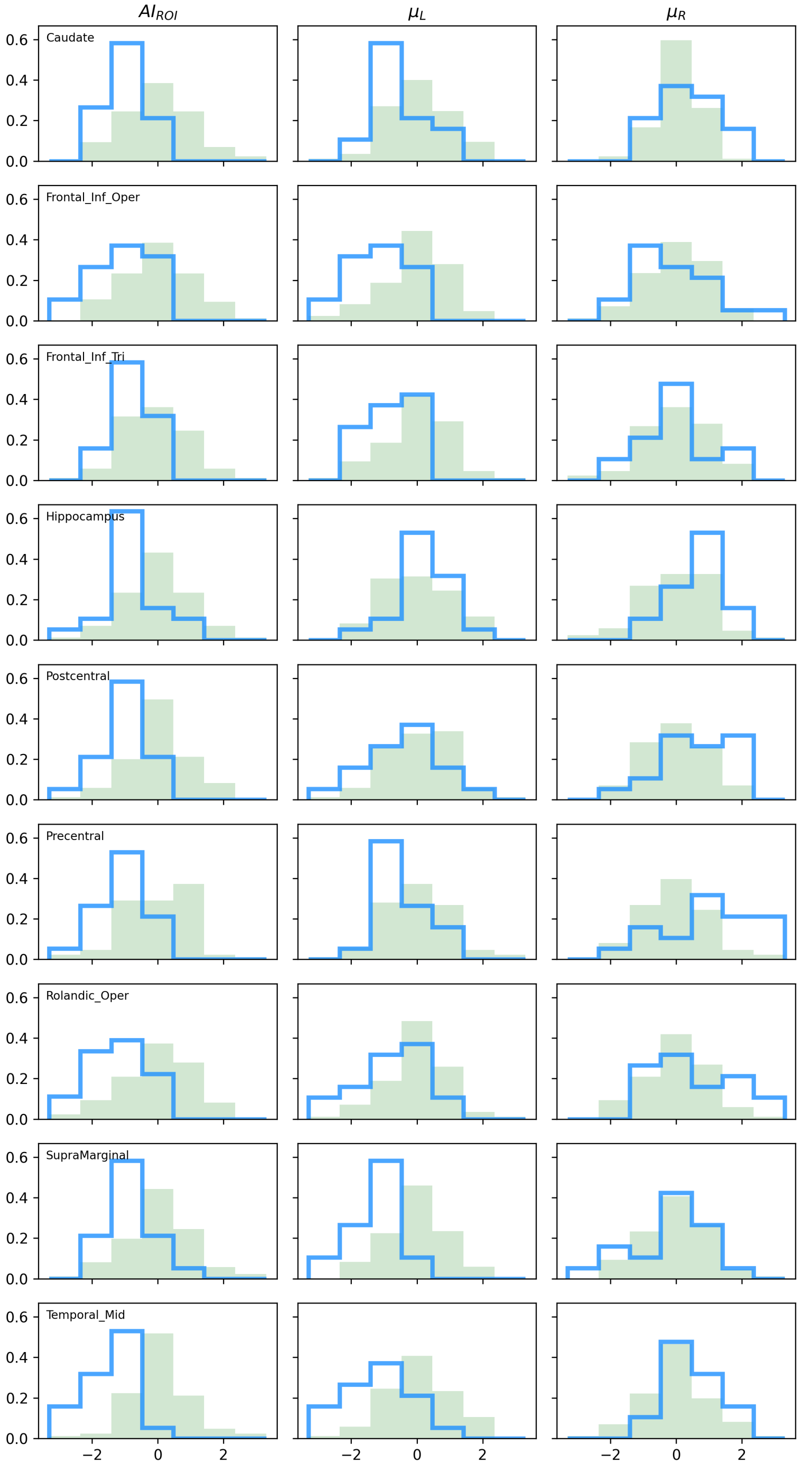

Supplementary Figure 2 ROI asymmetry index and z-scored mean uptake in left and right of the regions where AI_{ROI} was significantly different between iRBD (blue) and controls (filled green histogram).

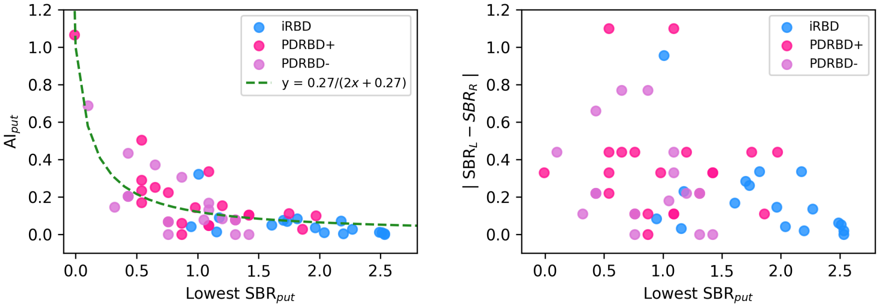

(a) (b)

Supplementary Figure 3 Lowest putamen SBR versus DAT asymmetry index (a) or absolute difference in putamen SBR (b). The green dotted line in (a) describes the resulting AI_{put} for a median difference between highest and lowest putamen SBR (0.27 for our PD patients), plotted as a function of lowest putamen SBR.

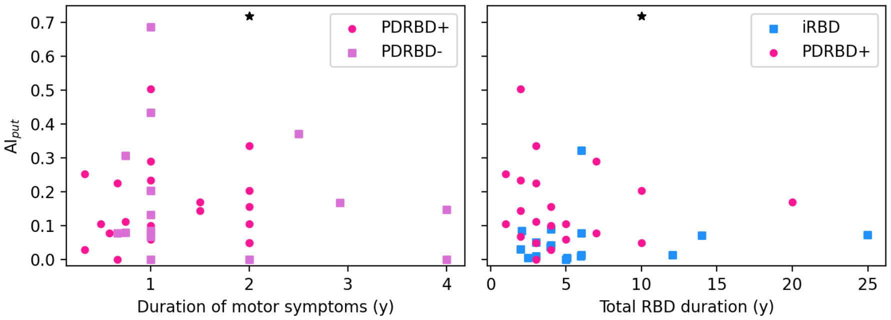

(a) (b)

Supplementary Figure 4 AI_{put} vs duration of motor symptoms for the PD groups (a), and total RBD duration for the RBD-positive groups (b). No correlation between either duration of motor or RBD symptoms was found for any of the groups ($p > 0.2$). A PDRBD+ outlier with $AI_{put} = 1.06$ has been marked with a black star.

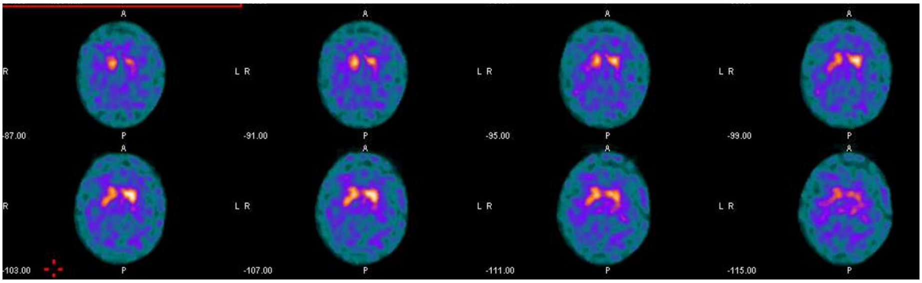

(a)

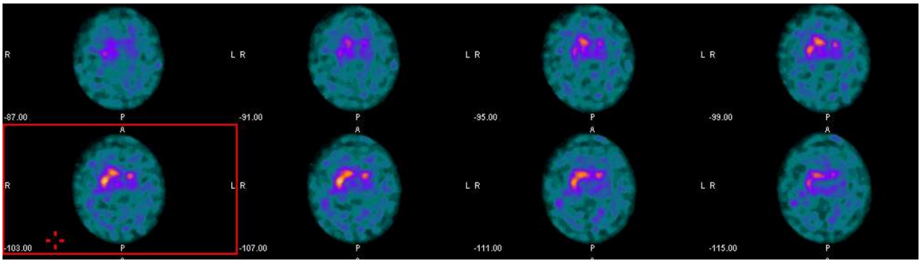

(b)

Supplementary Figure 5 DAT SPECT example snapshots for two PDRBD+ subjects. Symmetrically decreased striatal binding ((a), $\mathrm{AI}_{put} = 0.0$), and asymmetrically decreased binding ((b), $\mathrm{AI}_{put} = 0.5$).

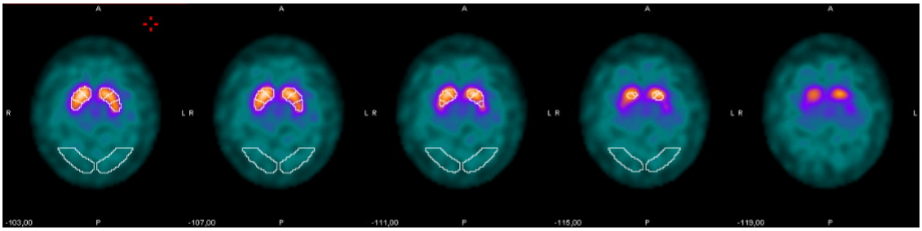

(a)

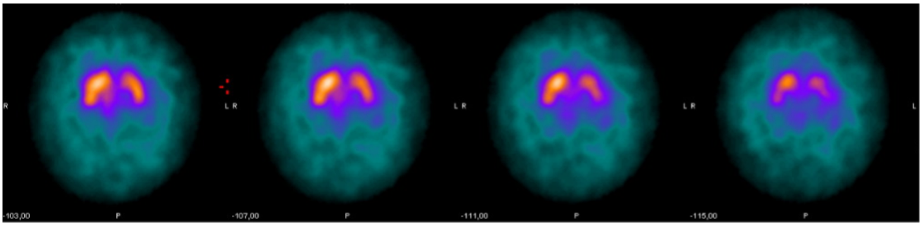

(b)

Supplementary Figure 6 DAT SPECT example snapshots for two iRBD subjects. Symmetrically decreased striatal binding ((a), $\mathrm{AI}_{put} = 0.0$), and asymmetrically decreased binding ((b), $\mathrm{AI}_{put} = 0.32$). The white overlays in the symmetric patient are simply a feature of the visualization program and did not play a specific role in our analysis.

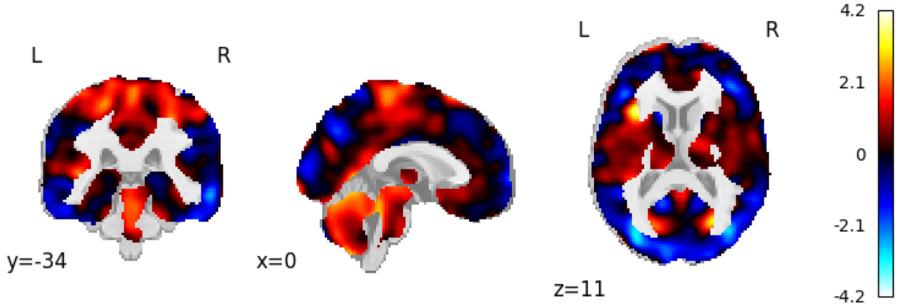

(a)

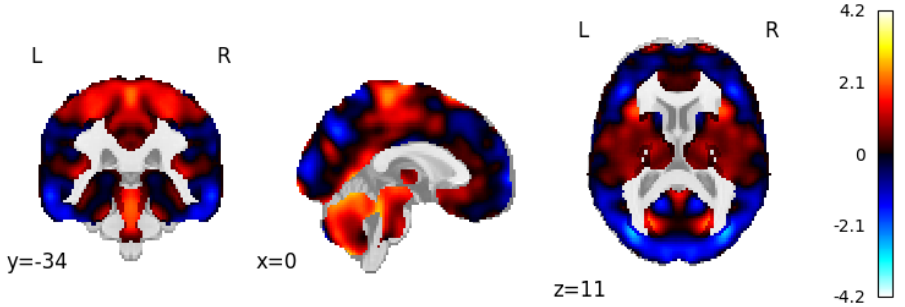

(b)

Supplementary Figure 7 Topographical map of the PDRP defined in [1] (a) and the mathematically symmetric version used in this work (b). The PDRP represents the relative glucose uptake of different regions of the brain in a PD patient, where low values represent areas with relative hypometabolism. A PDRP score represents how much the relative metabolism in an FDG-PET scan of a patient resembles the PDRP disease pattern. The voxel-wise Pearson correlation between the original and the symmetric pattern was $\rho = 0.92$.

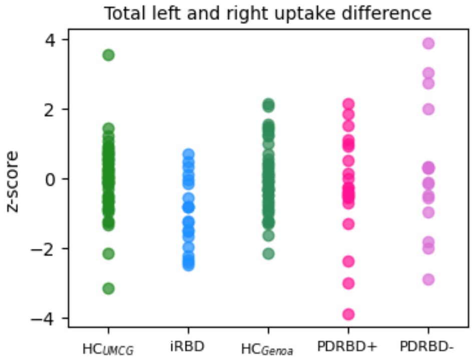

Supplementary Figure 8 Overall uptake difference between the left and right hemispheres, where the values have been z-scored to healthy controls from the same center. The iRBD patients showed a significantly lower uptake in the left and right hemisphere compared to healthy controls (HC) (mean -1.0 vs 0.0, $p = 0.006$), while the PD groups did neither differ compared to each other nor HC ($p \geq 0.4$).

References

[1] Teune, L. K. *et al.* Parkinson's disease-related perfusion and glucose metabolic brain patterns identified with pcasl-mri and fdg-pet imaging. *NeuroImage: Clinical* **5**, 240–244 (2014) .
